# Analysis of Factors Determining Serologic Response to Treatment of Early Syphilis in Adult Men

**DOI:** 10.3390/idr17030041

**Published:** 2025-04-27

**Authors:** Justyna Czarny, Damian Kadylak, Małgorzata Sokołowska-Wojdyło, Roman J. Nowicki

**Affiliations:** 1 Department of Dermatology, Venerology and Allergology, Medical University of Gdańsk, Mariana Smoluchowskiego 17 Street, 80-214 Gdańsk, Poland; 2 Department of Dermatology, Venereology and Allergology, University Clinical Centre, Mariana Smoluchowskiego 17 Street, 80-214 Gdańsk, Poland

**Keywords:** syphilis, treatment, sexually transmitted infections

## Abstract

**Background:** Syphilis is an infectious systemic disease that remains a public health threat, with an increasing incidence worldwide. Despite the availability of diagnostic tests and effective treatments, achieving a serological cure remains challenging for some patients. **Methods:** A retrospective cohort study of 130 male patients with early syphilis who attended the Department of Dermatology Venereology and Allergology in Gdansk was carried out between 2021 and 2024. This study assessed the rates of proper serological response and seroreversion of the VDRL test during the posttreatment follow-up period and analyzed selected factors influencing the achievement of these points. **Results:** The treatment outcomes were favorable; 96.15% of the patients achieved a proper serological response at a median of 1.54 months and seroreversion of the VDRL test within 18 months (median time = 7 months). A significantly greater proper serological response was observed in the primary and secondary syphilis patients than in the early latent syphilis patients (*p* = 0.005). A proper serological response was associated with age over 30 years (risk ratio (*RR*) = 1.381, *p* = 0.008) and VDRL baseline titers (≥1:32) (RR = 1.484, *p* = 0.005). The patients in the secondary or latent stage of early syphilis had a lower risk of seroreversion than those in the primary stage did (RR = 0.590, *p* = 0.030; *RR* = 0.560, *p* = 0.019, respectively). High titers at baseline (≥1:32) were also associated with a 30.8% reduced risk of seroreversion compared with lower titers (*RR* = 0.692, *p* = 0.038). **Conclusions**: These results suggest that age, syphilis stage, and titer level are significant predictors of the response rate. Based on these results, it is recommended that serological follow-up be concentrated within the first three months posttreatment, as this period accounts for the majority of treatment responses.

## 1. Introduction

Syphilis is an infectious systemic disease that remains a public health threat, with an increasing incidence worldwide.

According to a World Health Organization (WHO) report published in 2024, an estimated 8 million adults aged 15–49 years were infected with syphilis worldwide in 2022, and homosexual men and men who have sex with men (MSM) were disproportionately affected [1].

Based on data from the mandatory epidemiology surveillance system and Polish National Health Fund data, in 2023, the syphilis diagnosis rate in Poland was 7.93 per 100,000, and the reported number of syphilis cases nearly doubled from 2019 to 2023 [2].

Serological tests remain the basis for diagnosing syphilis and assessing the effectiveness of treatment. Serological monitoring after treatment is one of the key elements in patient management. Failure to obtain an appropriate serological response to treatment is an indication for further diagnostics, exclusion of reinfection, or exclusion of organ involvement, including neurosyphilis [3,4,5,6].

Although treatment of syphilis according to guidelines is highly effective, in some cases, it may be problematic to unequivocally confirm the cured status.

In early syphilis, a fourfold decrease in the nontreponemal test titer (NTTs), such as VDRL or RPR, within 12 months compared with the titer on the day of treatment is a laboratory indicator of the effectiveness of treatment. It is considered a proper serological response [3,4,5,6,7].

However, the time that a serologic response is obtained varies among patients. This study aimed to assess the rate of decline in serological response and assess the influence of selected factors on its success.

## 2. Materials and Methods

### 2.1. Study Design

A retrospective cohort study of patients who attended the Dermato-Venereology Outpatient Clinic in Gdansk (Poland) between May 2021 and October 2024 was carried out. Sociodemographic characteristics, clinical and sexual behavior data, and laboratory results were routinely collected.

In the study cohort, the probability of achieving favorable outcomes over time, including treatment response and serological negativization (seroreversion) in early syphilis, was estimated depending on selected factors, such as high-risk behaviors (e.g., MSM status, PrEP usage), HIV coinfection, syphilis staging, baseline VDRL titers, and treatment regimens.

### 2.2. Inclusion and Exclusion Criteria

We analyzed data from all patients who met the following criteria: (1) adult male patients diagnosed and treated for early syphilis during the 2021–2024 study period; (2) a reactive nontreponemal test VDRL (*Venereal Disease Research Laboratory*, VDRL) and treponemal tests (TPHA; *Treponema pallidum hemagglutination assay*) on the day of treatment; (3) therapy with a single dose of 2.4 MU benzathine penicillin (BPG) or doxycycline 100 mg twice daily for 14 days; and (4) serological monitoring after treatment consisting of VDRL determination on the day of treatment and every 3 months during follow-up visits.

Episodes of syphilis with nonreactive VDRL on the day of treatment and patients who did not attend follow-up monitoring or whose data were incomplete after treatment were excluded.

### 2.3. Characteristics of the Study Sample

The analysis was conducted on a cohort of 130 adult men aged 18–59 years who were diagnosed with early syphilis. The median age of the patients was 30.5 years (*Q*1 = 24.0, *Q*3 = 38.8 years).

### 2.4. Study Outcomes and Definitions

The primary objective of this analysis was to identify risk factors and favorable factors associated with the occurrence of proper treatment response and seroreversion of early syphilis among adult men. The stage of the disease was classified based on the time of infection acquisition, according to the CDC and IUSTI guidelines, and a duration of less than 1 year was considered early syphilis (primary, secondary, or early latent) [3,6].

The diagnosis of primary syphilis was made based on the presence of ulcers (single or multiple) or “chancre” in the genital or extragenital (rectal or oral) region in patients with syphilis diagnosis confirmed by serologic tests (reactive VDRL and TPHA tests). Secondary syphilis was diagnosed in patients with reactive VDRL and TPHA tests and concomitant maculopapular rash and/or mucous lesions, such as patches or condylomata lata and/or constitutional symptoms (fever, diffuse lymphadenopathy).

An asymptomatic patient with positive VDRL and TPHA test results and previous serologic tests for syphilis that confirmed positive results within 12 months of diagnosis was diagnosed with early latent syphilis.

Repeated syphilis infection was defined as a new episode of syphilis in an individual who had previously been treated and cured of the infection. This diagnosis was confirmed by observing a ≥4-fold increase in the VDRL from the previously recorded lowest value [3].

Follow-up visits after syphilis treatment were scheduled at 1, 3, 6, and 12 months after treatment. During these visits, the VDRL (*Venereal Disease Research Laboratory*, VDRL) titer was monitored. In our study, a proper serological response was defined as a ≥4-fold decrease (2-fold dilution) in the VDRL titer at 6–12 months after treatment for early syphilis. Seroreversion was defined as nonreactive VDRL in the follow-up period after treatment [3,6].

Patients whose VDRL titer increased ≥4-fold in the following period with exposure were considered to be reinfected.

Serological failure is defined as a less than 4-fold decrease in the NTTs (nontreponemal tests) 12 months after initial therapy for early syphilis [3,4,5,6,7].

### 2.5. Ethics

The Bioethics Committee of the Medical University of Gdansk approved this retrospective study (Protocol Number KB/521/2023, approved on 15 September 2023). 

### 2.6. Statistical Analysis

Statistical analysis was performed using a significance level of α = 0.05, corresponding to a 5% probability of making a Type I error, which was defined as incorrectly rejecting the null hypothesis when it was true.

The probability of achieving favorable outcomes over time, including treatment response and serological seroreversion in early syphilis, was estimated in the studied cohort via risk calculations (1 − *x*), where *x* represents survival. These probabilities were visualized via survival curves to illustrate the dynamic changes over time. Additionally, the number of patients at risk (at observation) and the cumulative number of events (patients achieved a proper serologic response or seroreversion) observed at specific time points were reported to provide context and ensure transparency regarding the data distribution.

The log-rank test was employed as the primary statistical method to compare risk curves between groups. In instances where the proportional hazards assumption was violated, alternative approaches were used to account for nonproportionality. Specifically, the Fleming–Harrington test was applied for comparisons involving more than two subgroups, whereas the Peto–Peto test was used for comparisons between two subgroups.

At the multivariable analysis stage, accelerated failure time (AFT) models were employed instead of the traditional Cox proportional hazards model because of the violation of the proportional hazard assumption. The AFT model offers an alternative approach that does not depend on the proportionality of hazards, allowing for direct modelling of the effect of covariates on survival time. To enable the interpretation of the coefficients directly in terms of risk, the regression coefficients were multiplied by “−1”. The AFT model’s distribution choice was determined by comparing competing distributions, specifically the Weibull, log-normal, and log-logistic distributions. The selection was based on the performance of the models concerning statistical criteria, including the Akaike information criterion (AIC), the log-likelihood values, and the χ^2^ test, which assesses the variability in response times explained by the covariates. The distribution that demonstrated the best fit (lowest AIC and log-likelihood) and highest explanatory power was chosen for the final analysis. Analyses were conducted via the *R* statistical language (version 4.3.3; R Core Team, Vienna, Austria, 2024).

## 3. Results

### 3.1. Characteristics of the Clinical and Epidemiological Parameters of Syphilis in the Study Cohort

Between May 2021 and October 2024, 130 male patients with early syphilis were diagnosed and monitored during the observation period.

Table 1 presents the clinical and epidemiological characteristics of the patient cohort, including demographic and behavioral factors (e.g., MSM status ad PrEP usage), stage of infection, treatment regimens, and follow-up outcomes.

Treatment outcomes were favorable, with 96.2% of patients receiving a proper therapy response within a median follow-up of 1.5 months (*IQR*: 1.3–3.0 months). However, seroconversion, defined as a nonreactive VDRL test, was achieved in only 59.2% of patients, with a median time to seroreversion of 4.9 months (*IQR*: 3.0–8.5 months).

### 3.2. Analysis of the Probability of Achieving a Serological Response and Seroreversion over Time

**Figure 1:**
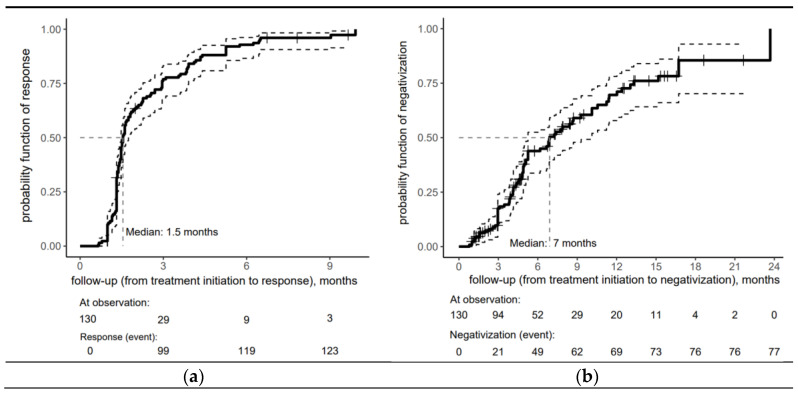
Risk curve for the overall cohort depicting (**a**) serological response and (**b**) seroreversion to early syphilis after treatment with an accompanying risk table (the solid lines represent the central estimate of the confidence interval, while the dashed lines indicate the lower and upper bounds. The crosses denote censored patients).

Figure 1 provides insight into the timing of a proper serological response (a) and seroreversion (b) after treatment for early syphilis within the cohort. One month after treatment initiation, the probability of a proper serological response was 10%, increasing sharply to 76.9% by three months. Half of the cohort achieved a proper serological response within 6 weeks of treatment initiation (the median time to respond was estimated at 1.5 months). By 9 months, the risk probability reached 96%, demonstrating that nearly all patients had responded to treatment by this time. The minimal increase after 6 months indicates that the treatment response had plateaued mainly, with only a tiny fraction of patients responding during later follow-up.

The risk curve for the overall cohort depicting seroreversion in early syphilis patients in Figure 1b shows the biphasic response pattern, with VDRL negativization achieved in the vast majority of patients within 18 months (median time = 7 months).

This rapid initial response (43.9% by 6 months) was followed by a moderate yet sustained response, with the risk probability reaching 69.6% at 12 months, reflecting the seroreversion of nearly 70% of the patients.

The trend in risk probability beyond 12 months reveals a deceleration in the rate of seroreversion, as evidenced by a more gradual increase in risk probability, which reached 85.5% at 18 months.

### 3.3. Analysis of the Influence of Selected Factors on Treatment Response: The Log-Rank Test

In the next step, an analysis of the risk for treatment response to early syphilis was conducted in subgroups based on various factors, including age, MSM status, PrEP use, HIV status, reinfection, disease stage, treatment (BPG or doxycycline), and baseline antibody titers.

**Figure 2:**
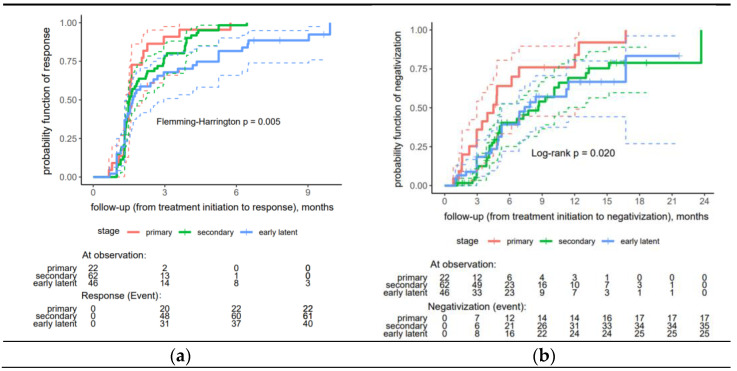
Risk curves stratified by stage for treatment response to early syphilis: (**a**) proper serological response, (**b**) seroreversion (negativization). In a Kaplan-Meier (KM) plot, the solid line, depicted in an appropriate color, represents the estimated a response probability for a specific group over time. The dashed lines, also in a matching appropriate color, indicate the 95% confidence interval (CI) for the response probability at each time point.

A significantly faster proper serological response was observed in the primary and secondary syphilis patients than in the early latent syphilis patients (*p* = 0.005) (Figure 2a).

The analysis found no significant difference between the remaining factors (Table S1—Supplementary Material). Regarding seroreversion, the patients in the primary stage of syphilis demonstrated significantly higher rates of negativization compared to those in the secondary and early latent stages (*p* = 0.020) (Figure 2b).

### 3.4. Evaluation of Determinants of Treatment Response in Early Syphilis Patients: A Multivariate Analysis

#### 3.4.1. Treatment Response

The Weibull accelerated failure time model included selected predictors (syphilis stage, HIV status, age, reinfection status, and treatment regimen), which were analyzed in subgroups to evaluate their effects on treatment response in early syphilis patients.

The results of the multivariate analyses (the Weibull accelerated failure time model) of the selected factors are presented in detail in Table 2.

The patients over 30 years had a 38.1% greater risk of a faster proper serological response than the younger individuals (risk ratio (*RR*) = 1.381, *p* = 0.008). Similarly, the patients with high titers (≥1:32) had a 48.4% increased risk of a faster response than those with lower titers (RR = 1.484, *p* = 0.005). Notably, the patients in the early latent stage of syphilis had a 47.4% lower risk of a faster response than those in the primary stage (*RR* = 0.526, *p* < 0.001). However, the patients with secondary syphilis were not significantly different from the patients with primary syphilis (RR = 0.767, *p* = 0.119).

Interestingly, there was no association between the risk of treatment response and other factors, including treatment regimen, HIV coinfection, and reinfection.

The overall model fit was highly significant at χ^2^ (10) = 47.62, *p* < 0.001, indicating that the covariates explained a substantial portion of the variability in response times.

#### 3.4.2. Seroreversion in Early Syphilis

A log-normal AFT model was used to evaluate the factors associated with the time to seroreversion of syphilis.

The analysis demonstrated that disease stage and titer level were significant predictors of the rate of seroreversion. The patients in the secondary stage of syphilis had a 41% lower risk of faster seroreversion than those in the primary stage (RR = 0.590, *p* = 0.030). Similarly, the patients with early latent syphilis had a 44% lower risk of faster seroreversion than those with primary syphilis (RR = 0.560, *p* = 0.019). High titers at baseline (≥1:32) were also associated with a 30.8% reduced risk of faster seroreversion compared with lower titers (RR = 0.692, *p* = 0.038).

The remaining factors associated with HIV coinfection, reinfection, and treatment regimens had no significant effect and were similar to those associated with proper serological response in our analysis (Table 2).

## 4. Discussion

Overall, the subjects included in our study had favorable outcomes, with a median time to proper serological response estimated at 1.5 months. This emphasizes the rapid effectiveness of therapy, and the first three months of serological follow-up largely determine the trajectory of the serological response.

In our practice, the first serological control is performed after 1 month, or, more precisely, after 7 weeks from the treatment injection, taking into account 3 weeks of treponemicidal activity of a single injection of benzathine benzylpenicillin [5,6,7,8].

Although the timing of the first serologic follow-up after treatment varies across guidelines, including 1 month (IUSTI), 3 months (BASHH), and 6 months (CDC), it appears that the 3-month follow-up after treatment provides valuable indications of the rate of subsequent serologic response and may facilitate decisions regarding the need for further follow-up [3,4,6].

The studies published thus far have assessed the influence of factors on the serological response; however, data on the influence of factors on seroreversion are scarce.

Our analyses assessed the two endpoints—proper serologic response and seroreversion—separately.

According to the guidelines, a decrease of at least 4-fold in the nontreponemal titer is a decisive factor in achieving a proper serological response and allows the end of observation, even if low titers are stable for a year [3,4,6,7]

This is related to the fact that the seroreversion percentage ranges from 13% to 44% and that patients with persistent high titers within 12 months are an indication for extended diagnostics and, by some authors, for puncture of cerebrospinal fluid [7,9,10].

This is the first study investigating the influence of selected factors on seroreversion and the response rate. The results suggest that primary syphilis and lower titer levels (up to 1:32) are important predictors of the speed of seroreversion in patients with early latent and secondary syphilis and those with high titers experiencing slower seroreversion.

Based on our analysis, patients who achieve proper serological response the fastest are those over 30 years of age, with high VDRL titers at baseline, and with primary or secondary syphilis.

High titers at baseline are associated with a faster proper serological response but slower seroconversion; ultimately, they are a factor in a favorable outcome.

Data in the literature indicate differences in the proportion of patients who achieve a serological response to treatment depending on the period of the disease and indicate that in late syphilis, there is a greater risk of treatment failure [10,11,12,13]. In our study, the study group included only people with early syphilis, but we also found some differences among these groups.

The symptomatic course of early syphilis (primary, secondary) was associated with a faster proper serological response to treatment than in the patients with early latent syphilis. Moreover, the patients with primary syphilis were significantly more likely to achieve seroreversion. A slower response in late latent syphilis patients has also been previously reported [11]. There are no known factors by which the involvement of the skin and mucous membranes contributes to the achievement of early clinical remission. However, one of the hypothetical factors may be earlier treatment in the case of a symptomatic course of the disease. Further research involving a larger patient cohort is necessary for a comprehensive prognostic assessment.

Another frequently assessed factor is the initial NTT (nontreponemal test) titer. Our study analyzed the VDRL titer from the day of treatment to baseline. Our results revealed that a faster treatment response for early syphilis was associated with higher (≥1:32) VDRL baseline titers than with lower titers.

A relatively high NTT titer, rapid plasma reagin (RPR 1:32), has also been shown to be associated with proper serological response in both early and late syphilis patients, according to previous studies in both HIV-negative and HIV-positive groups [11,12,14,15,16,17,18,19].

Another observation reported by Jinno et al. revealed that one of the strongest predictors of serological failure is an RPR titer ≤1:16 [15]. This finding is consistent with the observation that patients with low titers may more often fail to achieve a proper serological response, where a 4-fold decrease in the titer may be more challenging to detect in patients with low initial titers, which is often found in late syphilis [3,6,19]. The authors proposed that high VDRL titers may reflect a stronger immune response and a more remarkable ability to contain the infection [15,16].

Moreover, in our group of patients over 30, the probability of obtaining a proper serological response was greater than that in younger individuals. However, this did not significantly influence the achievement of seroreversion. Further studies are needed to confirm this correlation.

In our study, neither reinfection nor HIV coinfection significantly influenced the serological response to therapy.

Previous studies on persons living with HIV (PLWH) reported a slower serological response after treatment, which was associated with treatment failure [11,12,14]. A slower achievement of serologic remission in the HIV-positive group but a similar response rate in both groups after 2 years was reported by Gonzalez-Lopez et al. [12]. In the mentioned study, the researchers noted that antiretroviral therapy significantly reduces the time to achieve a response to syphilis treatment in PLWH [12].

A similar correlation was reported by Nieuwenburg et al., who reported that HIV-negative MSM are more likely to achieve an adequate serological response to treatment after 6 months than are HIV-positive MSM, but this difference is not apparent after 12 months, and an adequate titer response can be expected within 1 year; ultimately, HIV coinfection does not affect the outcome of syphilis treatment [20,21].

The heterogeneous results reported from studies assessing HIV coinfection as a factor determining serologic response could be due to differences in ART therapy, immunological status, and CD4 T-cell count.

A study conducted by Marchese V. et al. showed that there was no significant statistical difference in serological response to treatment in syphilis reinfection between PLWH and HIV-negative patients [22].

The above-mentioned study found that in PLWH, a baseline RPR ratio of >1:16 was associated with an increased risk of non-response. In contrast, the assessment conducted 12 months after treatment did not show a significant difference in achieving serological response, indicating that patients with HIV/AIDS show a slower serological response to treatment, which has been highlighted in other studies [20,21,22].

Finally, no differences in serological cure rates were observed between treatment regimens (1-dose BPG vs. doxycycline) in our cohort.

Our results are consistent with those of other studies in which the risk of treatment response was not associated with treatment regimens (BPG vs. doxycycline) in early and late syphilis patients. However, the results for late latent syphilis patients should be interpreted with caution because of the limited sample size treated with doxycycline at this stage [14,23,24].

Our study has several limitations. First, due to the study’s retrospective nature, the patients had different follow-up durations. Second, our sample group included only male patients, mostly MSM, who were patients at the STD clinic. Therefore, our results are only representative of this specific population. Further study limitations include the relatively small group treated with doxycycline, which is associated with low statistical impact. Another limitation is the non-specificity of the VDRL test. False-positive VDRL results may result from a technical error or the presence of antiphospholipid autoantibodies (biological false positivity) that infectious and non-infectious agents can induce. Our study included patients whose diagnosis of syphilis was supported by reactive TPHA and nontreponemal VDRL, essentially eliminating non-specific VDRL reactivity from other causes.

Repeated syphilis infection was defined as a new episode of syphilis in an individual who had previously been treated, and the diagnosis was confirmed by observing a ≥ 4-fold increase in the NTTs from the previously recorded lowest value. Therefore, we cannot distinguish these patients from those who experienced relapse due to treatment failure.

Moreover, we cannot ensure that the patient did not take antibiotics between follow-up visits because of independent infections whose antimicrobial effects may affect *Treponema pallidum*. Finally, this study presents data collected from a single center.

## 5. Conclusions

These results suggest that the syphilis stage and titer level are significant predictors of the speed of response. In contrast, other factors, such as reinfection status, HIV coinfection, PREP use, and treatment type, may warrant further investigation in more extensive studies.

Our results may be helpful in clinical practice for patients who do not achieve remission at 3 months of follow-up, as they may require special attention.

## Figures and Tables

**Table 1:** Clinical and epidemiological parameters characterizing the profile of patients, disease severity, and treatment and follow-up results.

Characteristic	Total (n = 130)
Age	30.5 ^a^
MSM	108 (83.1%)
PREP usage	6 (4.6%)
HIV	20 (15.4%)
Reinfection	35 (26.9%)
Syphilis stage	
early latent	46 (35.4%)
primary	22 (16.9%)
secondary	62 (47.7%)
Treatment:	
1BPG ^b^	122 (93.9%)
Doxycycline	8 (6.2%)
Titer at baseline	
up to 1:16	59 (45.4%)
1:32 or above	71 (54.6%)
Response to treatment (at least 4*x* decrease in titers)	125 (96.2%)
Follow-up response to treatment, months	1.5 (1.3, 3.0) ^a^
Seroreversion (VDRL)	77 (59.2%)
Following disease to treatment, months	4.9 (3.0, 8.5) ^a^

^a^—Median (*IQR*). ^b^—1 BPG-1-dose benzathine penicillin G intramuscular.

**Table 2:** Multivariate analysis: a Weibull accelerated failure time (AFT) model: factors associated with serological response.

Characteristic	Proper Serologic Response		Seroreversion	
	RR	*p*-Value	RR	*p*-Value
Aged over 30 years vs. below	1.381	0.008	1.100	0.583
Baseline titers ≥1:32 vs. <1:32	1.484	0.005	0.692	0.038
Early latent vs. primary syphilis	0.526	<0.001	0.560	0.019
Secondary vs. primary syphilis	0.767	0.119	0.590	0.030
Reinfection, yes vs. no	0.771	0.124	0.966	0.866
Doxycycline vs. BPG	0.704	0.150	1.153	0.669
HIV, yes vs. no	1.151	0.392	1.076	0.735

RR—Risk ratio.

## Data Availability

All data generated during this study are included in this article or as Supplementary Files.

## Supplementary Materials

The following supporting information can be downloaded at https://www.mdpi.com/article/10.3390/idr17030041/s1, Table S1: Factors determining proper serological response (univariate analysis).

## Abbreviations

The following abbreviations are used in this manuscript:

ART	Antiretroviral therapy.
BPG	Benzathine penicillin.
TPHA	*Treponema pallidum* hemagglutination assay.
VDRL	Venereal Disease Laboratory Research.
