# Supplementary material for: Analysis of Factors Determining Serologic Response to Treatment of Early Syphilis in Adult Men
Source: Infect Dis Rep. 2025 Apr 27;17(3):41. doi: 10.3390/idr17030041 (PMC12101400; doi:10.3390/idr17030041)
Supplement: Supplementary file 1 [file idr-17-00041-s001.zip › idr-3519362-supplementary.pdf]

Supplementary material

Table S1.

Factors determining proper serological response (univariate analysis)

Characteristic	Proper Serologic Response	
		p-value
**Aged over 30 years vs below**	Log-rank test	0.764
**Primary syphilis vs early latent**		0.005
**Secondary syphilis vs early latent**		0.005
**Reinfection, yes vs no**		0.104
**Doxycycline vs BPG**		0.452
**HIV, yes vs no**		0.625
**MSM, yes vs no**		0.243
**PREP, yes vs no**		0.325
**Baseline titres ≥1:32 vs <1:32**	Peto-Peto test	0.300
